# Mobile Apps to Improve Health Parameters in Healthy Adults: Systematic Review

**DOI:** 10.2196/66881

**Published:** 2026-01-16

**Authors:** Gaia Leuzzi, Mirko Job, Carola Cosentino, Riccardo Testa, Aldo Scafoglieri, Marco Testa

**Affiliations:** 1 Department of Neurosciences, Rehabilitation, Ophthalmology, Genetics, Maternal and Child Health, University of Genoa, Campus of Savona, Savona, Italy, 39 019 219 45703; 2 Department of Physical Education and Rehabilitation, Experimental Anatomy Research Group (EXAN), Vrije Universiteit of Brussel (VUB), Brussels, Belgium; 3 Department of Neurosciences, Rehabilitation, Ophthalmology, Genetics, Maternal and Child Health, University of Genoa, Genova, Italy; 4 Department of Electrical, Electronics, Telecommunication Engineering and Naval Architecture, University of Genoa, Genova, Italy

**Keywords:** active aging, diet, exercise, healthy ageing, health apps, mobile apps, physical activity

## Abstract

**Background:**

Recently, mobile health and mobile apps have been proposed as a potential tool to improve different outcomes (eg, daily steps, blood glucose) in both people with and without chronic conditions. In particular, healthy people could benefit from these tools by improving health variables and for prevention. Previous evidence investigated different types of health interventions adopting apps in various settings and populations, but evidence of their effectiveness is still unclear.

**Objective:**

The aim was to assess the effectiveness of mobile apps in improving health variables (eg, daily steps, maximal aerobic capacity) in healthy adults, involving an intervention regarding physical activity, diet, or their combination thereof. Evidence would suggest if apps could be effectively adopted in health interventions aiming toward prevention.

**Methods:**

A systematic review was performed using Medline via PubMed, Cochrane Library—CENTRAL, and Embase. Only randomized controlled trials comparing the same intervention provided with and without a mobile app or a treatment and a mobile app compared with the treatment only were included in this systematic review. The Risk of Bias tool 2.0 was used to assess the risk of bias, and the GRADE (Grading of Recommendations, Assessment, Development and Evaluation) was adopted for rating the certainty of evidence.

**Results:**

Considering studies up to June 2025, only 2 studies were included in the review of mobile apps for physical activity, and none were included for mobile apps for diet and none for mobile apps for physical activity and diet combined. The quality of evidence of the 2 studies included was low due to a high risk of bias, several missing data, and deviation from the original interventions, suggesting a scarce rigor in the methodology adopted. Therefore, mobile apps’ effectiveness in improving diet, physical activity, or their combination cannot be assessed.

**Conclusions:**

Despite the widespread use of mobile apps for health and the large number of relative publications, the results of this systematic review did not allow us to ascertain the effectiveness of mobile apps for health, but they provided fundamental insights for future research. Hence, it is not possible to state if apps for health might be used as supporting tools for health interventions aiming toward prevention and health improvements in healthy people. There is an urgent need to develop stronger evidence of apps’ effectiveness in addressing different populations and types of interventions for different health domains.

## Introduction

Promoting active aging [1] requires new strategies to reach different populations in a feasible and effective way to help people improve their health status [2,3]. For this reason, the use of technology interventions is getting paramount attention to help people improve their health variables [4], identified as both clinical (eg, blood pressure, weight) and nonclinical outcomes (eg, daily activity or sleep) [5,6]. The attention to the use of technology-supported health interventions is due to the ease of the use of mobile devices, their portability, their quality-price ratio [7], and the quantity of information they can provide with good data storage and live data analysis [8].

The World Health Organization describes this type of app as mobile health app (mHealth), defining it as “medical and public health practices supported by mobile devices, such as mobile phones, patient monitoring devices, personal digital assistants, and other wireless devices” [9]. Wireless devices can be fitness trackers, smartwatches, and smartphones, which makes it easy to collect several types of data (ie, number of daily steps, macronutrients, sleep, and stress level) from different health-related spheres, such as physical activity and diet [10,11], automatically (ie, using a wearable device [12]), through user’s action or both. In particular, to do so, smartphones allow for downloading different types of apps, including health ones. These apps can be focused on just 1 health-related sphere [13,14] or a combination thereof [15].

Due to their easy use and versatility, people with and without diseases can benefit from mHealth apps to prevent or treat different conditions [16,17]. In line with that, different studies have been carried out in the last few years to test the effectiveness of apps for health on people with [18] and without diseases [19]. Unfortunately, the quality of the studies is low, and the results are often controversial [20], thus not allowing for definitive results on this topic. Moreover, to understand the effect of apps for health, one should compare the effectiveness of an intervention provided with and without the adoption of an app, while most of these studies adopt apps as baseline treatment with different interventions as adjunctive therapy, therefore testing the effectiveness of the adjunctive therapy rather than the app one [21,22]. Furthermore, mobile apps were chosen as mHealth to be investigated, as they are one of the most adopted technologies worldwide in health contexts [23], and those targeting fitness, nutrition, and healthy living are widely diffused [24].

Hence, the main purpose of this systematic review is to analyze the effectiveness of mobile apps in improving healthy adults’ (ie, >18 years old) health variables, analyzing only randomized controlled trials (RCTs) that compare the same intervention with and without this technology in physical activity, diet, and a combination thereof.

## Methods

The protocol of this systematic review was created and submitted to PROSPERO [25] (CRD42023485803). Furthermore, PRISMA (Preferred Reporting Items for Systematic Reviews and Meta-Analyses; Checklist 1) [26] guidelines and PRISMA-S (Preferred Reporting Items for Systematic Reviews and Meta-Analyses Literature Search Extension; Checklist 2) [27] were followed to report this review.

### Deviations from the Protocol

The protocol initially restricted inclusion to English-language studies. During screening, it became clear that this would underrepresent available evidence and introduce potential language bias. We therefore expanded the criteria to include studies in other languages when reliable translation or accurate data extraction was possible. This deviation was made to enhance the review’s completeness and international representativeness, while maintaining all other methodological criteria.

### Study Objective

The main objective of this systematic review was to analyze the effectiveness of mobile apps for physical activity and diet to improve healthy adults’ health variables. The research question was as follows: are mobile apps effective in improving health-related variables in healthy adults? To address this topic, three different options were investigated: (1) mobile apps for physical activity, (2) mobile apps for diet, and (3) mobile apps for physical activity and diet. The main outcomes listed hereafter correspond to each of the abovementioned points: (1) physical activity variables (eg, daily steps, moderate-to-vigorous physical activity), (2) diet variables (eg, weight, BMI), and (3) physical activity and diet variables as mentioned above.

### Eligibility Criteria

For this systematic review, studies were considered eligible if they were published RCTs. No limitations on publication time were set, and RCTs published online until June 3, 2025, were included. Systematic reviews, reviews, meta-analyses, single-case studies, case series, observational studies, books, documents, guidelines, reports, and conference abstracts were excluded. Gray literature, systematic reviews, and meta-analyses were consulted, but not considered eligible, to find useful studies.

We included all those studies that involved healthy adult participants (>18 years old), with no cognitive impairments, musculoskeletal or neuromotor diseases, chronic conditions (eg, diabetes, hypertension), obese (ie, BMI≥30), or pregnant women. Interventions were considered eligible if a mobile app was used as an intervention to improve variables related to physical activity, diet, or their combination or only as a supportive technology to a specific intervention. No limitations on time or sessions of interventions were set. Mobile apps, including automatic or self-reported data collection and the use of a wearable device, were taken into account. Conversely, we excluded interventions with non-standalone apps, using high-cost sensors, exergames, nonmobile monitoring systems, robotics systems, or just clinician’s telemedicine. Moreover, studies that did not precisely describe the health conditions of their population were excluded.

### Search Strategy

Three scientific databases were sought for the study research: Medline via PubMed, Cochrane Library—CENTRAL, and Embase. They were chosen as they are reported as mandatory by the *Cochrane Handbook for Systematic Reviews of Interventions* [28].

The literature search was performed on the databases up to June 3, 2025, and the results were later merged into a single file to be subsequently uploaded onto Covidence [29], where the automatic duplicate detection was conducted. Specific search strings were created for the 3 databases, mixing Boolean operators (ie, AND, OR), MeSH terms, and keywords. The research strategy is reported below.

- Mobile apps and physical activity and RCT: ((mHealth) OR (m-health) OR (“mobile health”) OR (“mobile application”) OR (“mobile app”) OR (“smartphone application”) OR (“smartphone app”) OR (apps) OR (smartphone) OR (“Mobile Applications”[Mesh]) OR (“Smartphone”[Mesh])) AND ((fitness) OR (“physical exercise”) OR (“physical fitness”) OR (“fitness behavior”) OR (“Physical Fitness”[Mesh]) OR (“Exercise”[Mesh]) OR (pedometer) OR (steps) OR (exercise) OR (“training exercise”) OR (“heart rate variability”) OR (“Heart rate”) OR (“Heart Rate”[Mesh])) AND ((single blind) OR (double blind) OR (trial) OR (random*) OR (randomized) OR (randomized controlled))

- Mobile apps and diet and RCT: ((mHealth) OR (m-health) OR (“mobile health”) OR (“mobile application”) OR (“mobile app”) OR (“smartphone application”) OR (“smartphone app”) OR (apps) OR (smartphone) OR (“Mobile Applications”[Mesh]) OR (“Smartphone”[Mesh])) AND ((diet) OR (“calorie counter”) OR (“calorie counting”) OR (calorie) OR (“calorie intake”) OR (diet) OR (“Diet”[Mesh]) OR (dieting) OR (“weight loss”) OR (“weight loss”[Mesh]) OR (“Weight Reduction Programs”[Mesh])) AND ((single blind) OR (double blind) OR (trial) OR (random*) OR (randomized) OR (randomized controlled))

### Selection Process

Regarding the first research question, 2 researchers (GL and MJ) manually and independently screened titles and abstracts of the retrieved papers and evaluated them against the inclusion criteria. At the same time, for the second research question, 2 researchers (GL and RT) followed the same procedure. For the third research question, studies were identified among the papers selected by the above-described screening. The eligibility of the studies was then agreed upon through a consensus meeting between the 2 authors of each review and, in case of disagreement, a third researcher (CC) was consulted to reach a final decision. Afterward, the full texts of the selected papers were further screened against the inclusion criteria following the same process.

### Data Collection

Two researchers (GL and MJ and GL and RT) proceeded blindly and independently to extract specific data from each study such as authors, year of publication, country, intervention setting, study design, total number of participants, number of participants for each experimental group, mean age of the participants and standard deviation (if available), number of female and male participants, type and timing of intervention sessions for both experimental groups, number and timing of follow-ups, outcomes, key conclusions, and eventually even a researcher’s comment on each study. Moreover, all data available in each study were extracted and reported, such as mean, median, IQR, SD, number of follow-ups, and data registered at each follow-up. In case of missing data, authors were contacted.

### Data Items

The most relevant characteristics of the selected studies are summarized in Table 1. According to our research questions, the outcomes of this systematic review are grouped as follows: (1) physical activity outcomes, (2) diet outcomes, and (3) physical activity and diet outcomes. No limits were identified for the reporting of any outcome. In case of missing data, authors were contacted.

**Table 1.** Study characteristics.

Study characteristics	Zongpa et al [30] (2020)	Muntaner-Mas et al [31] (2021)
Total number of participants	47	66
App name	Take a Walk	Vidahora
Type of intervention	App+diet indications	App
Type of control	No app+diet indications	No app
Primary outcome	VO_2_ max^a^ + HRV^b^ + FBG^c^ + adherence	Weigh + waist and hip circumference + 20-m shuttle run test + handgrip + standing long jump test + 4 × 10 m shuttle run test + sit and reach + IFIS^d^
Follow-ups	Week 4	Week 9
Intervention sessions and duration	6 reminders/day, 1 for every working hour	Free

a VO_2_ max: maximal aerobic capacity.

b HRV: heart rate variability.

c FBG: fasting blood glucose.

d IFIS: International Fitness Scale.

### Risk of Bias Assessment

The risk of bias (RoB) assessment was performed, independently and blindly, by 2 researchers for each study (GL and MJ or GL and RT, respectively) following the Revised Cochrane Risk of Bias tool 2.0 (RoB 2.0) [32] for RCTs or the Rob 2 CRT for cluster-randomized controlled trials [33]. This tool aims at assessing the RoB specifically for 5 domains: “Risk of bias arising from the randomization process,” “Risk of bias due to deviations from the intended interventions,” “Risk of bias due to missing outcome data,” “Risk of bias in measurement of outcome,” and “Risk of bias in selection of the reported results.” Consequently, an overall RoB for the study is provided. Domains and studies can be classified at low, moderate, or high RoB. The tool also allows one to indicate “no information” as an answer for each item of every domain and, in this case, it would often be considered at high RoB. A third researcher (CC) was contacted in case of disagreement to reach a consensus.

### Statistical Analysis

Data from each study were extracted and reported, and a descriptive statistic was performed. For intergroup comparisons, the mean, SD, and/or mean differences for pre- and posttreatment conditions were reported. Additionally, the 1- or 2-tailed *t* tests for normally distributed data and the Mann-Whitney *U* test for nonnormally distributed data were also reported if performed in the studies. All statistical analyses were performed using the Jamovi statistical software [34].

### Quality of Evidence

To perform the quality of evidence assessment, the GRADE (Grading of Recommendations, Assessment, Development and Evaluation) [35] approach was used via the GRADEpro GTD tool. This tool helps assess both the certainty of evidence and the strength of recommendations. The evaluation process took into account 5 different domains: risk of bias, imprecision (eg, sample size, confidence intervals), inconsistency (eg, heterogeneity), indirectness (eg, eligibility criteria against actual studies included), and publication bias (eg, bias in results publication).

## Results

### Study Selection

**Figure 1.**
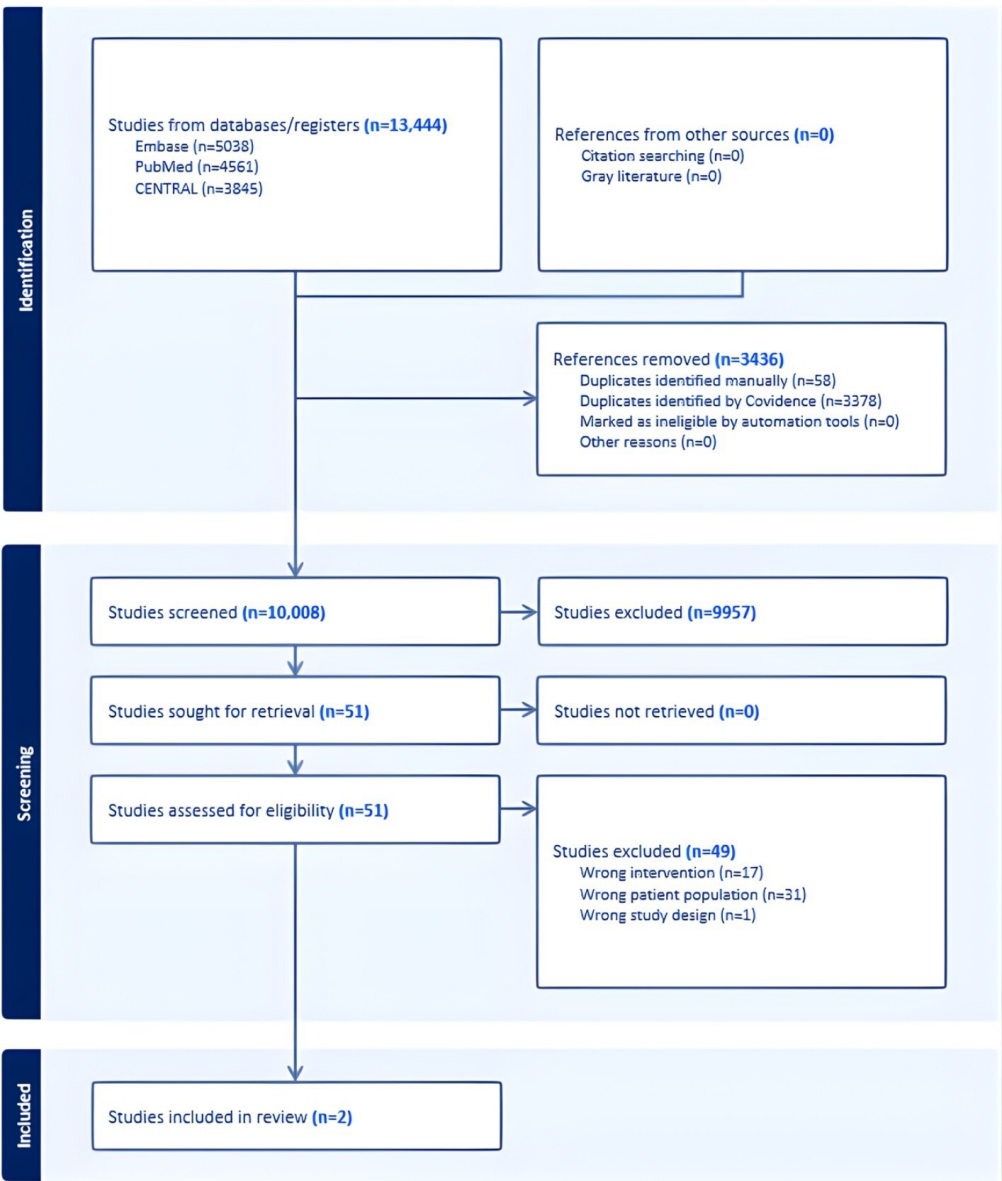
PRISMA (Preferred Reporting Items for Systematic Reviews and Meta-Analyses) flow diagram for mobile apps for physical activity.

The literary search process for the first review identified a total of 13,444 studies. Duplicate removal eliminated 3436 studies, leaving a total of 10,008 studies to screen. After applying the inclusion and exclusion criteria to titles and abstracts, 51 studies were left [19,30,31,36-82]. Full-text studies were read independently by 2 researchers (GL and MJ), and in due course, another 49 papers were excluded [19,36-82], resulting in the final inclusion of 2 studies for further analysis [30,31]. The complete research process is graphically displayed in Figure 1, and the reasons for exclusions are reported in Multimedia Appendix 1. Multimedia Appendix 2 reports the complete research processes of the other 2 research questions.

**Figure 2.**
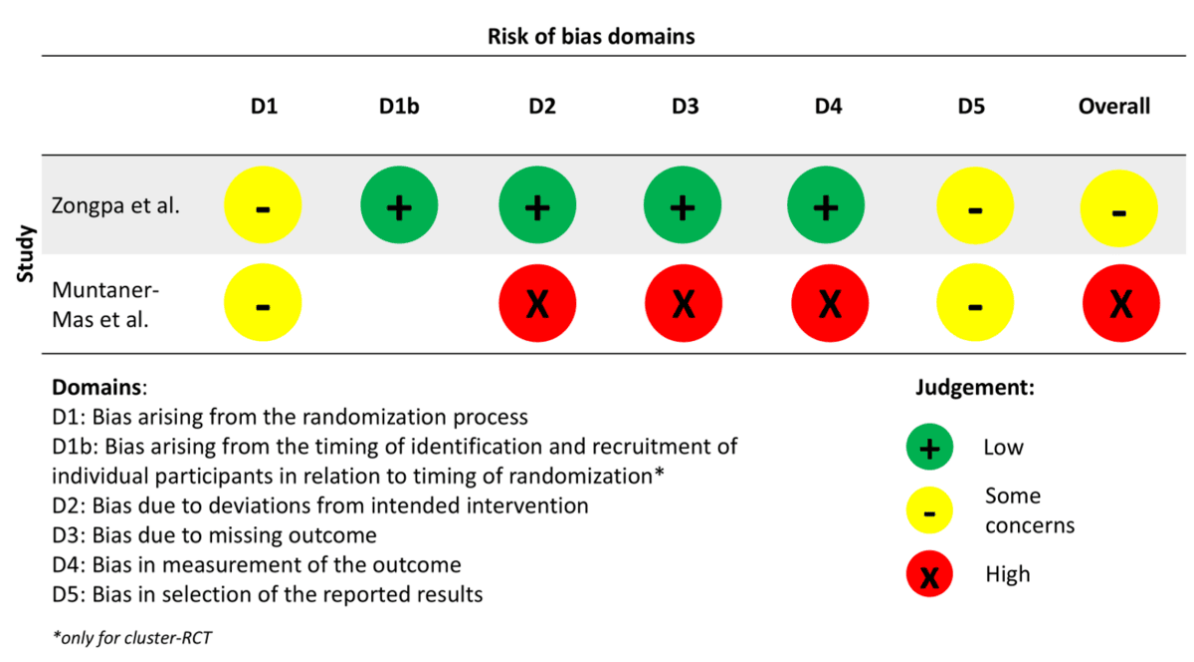
Risk of bias (RoB) assessment for randomized controlled trial (RCT) and cluster-RCT [30,31].

Regarding the second and third questions (ie, mobile app for diet, and physical activity and diet), unfortunately, no studies were included. The selection processes are shown in Multimedia Appendix 2.

### Study Characteristics

Among the 2 studies included in the final analysis, the first was a cluster RCT published in 2020 [30], and the second one was an RCT published in 2021 [31]. Both had only 1 intervention group and 1 control group [30,31]. The time of the intervention ranged from 4 to 9 weeks. The countries of study development were India [30] and Spain [31]. The studies’ characteristics are indicated in Table 1. The first study [30] involved 47 participants, and the intervention consisted of giving a smartphone app (Take a Walk) to remind them to perform a few minutes of walking at regular intervals. The intervention group received “walk breaks” reminders each hour of work for a total of 6 reminders a day. The intervention lasted 4 weeks. Both intervention and control groups received an indication to follow a standard diet of 2300 Kcal/day. Physical activity–related variables were evaluated using different tests or variables, such as the fasting blood glucose, VO_2_ max (maximal aerobic capacity), heart-rate variability, and adherence to walking breaks.

In the second study [31], involving 66 participants, the intervention group was provided with an app (Vidahora) to be used for 9 weeks. Participants were free to use the app whenever they wanted and were only advised to perform at least 10 minutes of physical activity 3 times a day. The second study assessed physical activity–related variables using several performance tests, anthropometric tests, and a questionnaire. Anthropometric tests were weight, hips, and waist circumferences. Performance tests were 20-m shuttle run test, handgrip test, standing long jump test, 4×10 m shuttle run test, and sit and reach test. The questionnaire used was the International Fitness Scale [83].

The characteristics of both studies are reported in Table 1, while the studies’ outcomes are reported in Table 2.

**Table 2.** Physical activity-related outcomes.

Authors (app used) and test	Baseline		Post-intervention	
	Intervention group	Control group	Intervention group	Control group
Zongpa et al [30] (Take a Walk)				
VO_2_ max^a^ (mL/kg/min), median (IQR)	45.3 (39.0-52.3)	36.0 (36.0-41.2)	47.6 (39.6-55.9)	37.5 (35-40.3)
HRV^b^				
Time domain, median (IQR)				
SDNN^c^ interval (ms)	52.7 (51.4-53.4)	49.1 (44.8-52.1)	54.5 (52.5-60.2)	49.4 (48.1-50.6)
RMSSD^d^ interval (ms)	52.4 (48.2-54.2)	54.2 (52.4-55.8)	58.1 (57.4-58.6)	55.1 (53.2-55.7)
NN50^e^ (beats)	129.4 (129.1-129.7)	129.7 (128.9-129.2)	130.3 (129.6-132.1)	126.2 (123.8-127.9)
pNN50^f^ (%)	37.4 (37.3-37.6)	37.2 (37.0-37.2)	39.0 (38.2-39.2)	35.3 (34.2-37.1)
Frequency domain, median (IQR)				
VLF^g^ (ms^2^/Hz)	103.00 (101.0-105.0)	103.0 (90.0-103.0)	107.0 (102.0-112.0)	90.1 (84.7-103.1)
LF^h^ (ms^2^/Hz)	981.0 (972.0-988.2)	984.3 (983.6-985.7)	986.5 (978.0-996.0)	976.0 (962.0-984.0)
HF^i^ (ms^2^/Hz)	970.0 (958.0-984.0)	981.5 (965.0-984.0)	986.0 (973.0-994.5)	975.0 (962.0-982.0)
LF/HF^j^ (%)	1.02 (0.98‐1.04)	1.0 (0.96‐1.04)	1.0 (0.9‐1.0)	1.0 (0.98‐1.04)
Nonlinear index, median (IQR)				
SD1^k^ (ms)	37.2 (36.1-38.3)	22.1 (22.0-25.1)	41.1 (38.2-44.1)	35.8 (34.5-37.7)
SD2^k^ (ms)	55.0 (51.8-59.7)	33.5 (30.7-33.5)	88.6 (72.0-88.6)	59.4 (57.6-61.1)
SD1/SD2 (%)	1.5 (1.2-1.8)	1.5 (1.2-1.5)	2.1 (1.9-2.2)	1.7 (1.3-2.0)
FBG^l^ (mmol/dL), median (IQR)	89.0 (78.0-93.2)	87.0 (81.0-88.0)	83.0 (72.0-86.0)	87.0 (81.0-92.1)
Adherence	NA^m^	NA	NA	NA
Muntaner-Mas et al [31] (Vidahora)				
Weight (kg), mean (SD)	65.1 (12.1)	65.1 (13.7)	65.7 (12.1)	65.5 (13.8)
Waist circumference (cm), mean (SD)	77.1 (9.9)	81.0 (11.8)	76.0 (11.7)	80.2 (11.2)
Hip circumference (cm), mean (SD)	96 (8.8)	96.3 (9.0)	93.3 (9.1)	95.7 (10.2)
20-m shuttle run (laps), mean (SD)	6.6 (3.2)	5.8 (3.4)	7.7 (2.8)	5.7 (3.3)
Handgrip strength (kg), mean (SD)	30.8 (8.0)	28.9 (7.9)	32.1 (9.0)	28.2 (9.1)
Standing broad jump (cm), mean (SD)	155.4 (35.2)	146.0 (31.0)	169.7 (35.9)	150.5 (29.4)
4×10 m shuttle run (sec), mean (SD)	11.4 (1.3)	11.7 (1.4)	11.3 (1.3)	12.0 (1.5)
Sit-and-reach (cm), mean (SD)	19.9 (8.9)	20.2 (8.9)	21.6 (9.7)	21.7 (8.3)
General physical fitness, mean (SD)	3.2 (0.7)	3.3 (0.9)	3.6 (0.6)	3.2 (0.9)
Cardiorespiratory fitness, mean (SD)	2.8 (1.0)	2.8 (1.1)	3.1 (1.0)	2.7 (1.1)
Muscular fitness, mean (SD)	3.2 (0.7)	0.1 (1.1)	3.4 (0.7)	3.2 (1.0)
Speed-agility, mean (SD)	3.3 (0.7)	3.3 (1.0)	3.5 (0.7)	3.3 (1.0)
Flexibility, mean (SD)	2.8 (0.9)	2.8 (1.2)	3.1 (1.2)	2.9 (1.1)

a VO_2_ max: maximal aerobic capacity.

b HRV: heart rate variability.

c SDNN interval: standard deviation of NN intervals.

d RMSSD: root mean square of successive RR interval differences.

e NN50: successive RR intervals that differ by more than 50 ms.

f pNN50: percentage of successive RR intervals that differ by more than 50 ms.

g VLF: very low frequency of power.

h LF: absolute power of the low-frequency band (0.04-0.15 Hz).

i HF: absolute power of the high-frequency band (0.15-0.4 Hz).

j LF/HF: ratio of LF-HF power.

k SD1 and SD2: Poincaré plots perpendicular to line of identity.

l FBG: fasting blood glucose.

m NA: not available but requested to authors.

### App’s Characteristics

Information about the Take a Walk app was limited; it was described as a simple Java–based Android app that allowed participants to set personalized reminders for walking, including customizable times and data. Participants were only required to manually set when to receive the reminders to walk.

The Vidahora app was made of 4 different sections: the first section dedicated to a quiz about healthy habits, the second section dedicated to the challenges for improving different physical activities’ components via suggested video exercises (eg, strength, aerobic exercise, yoga), the third section hosted an artificial intelligence–assisted chatbot that could ask the participant about progress in a friendly way, and the last section was for setting the user data (eg, username, personal data). Badges for achievements were also present, as well as individual and community challenges with daily or weekly aims. Participants in the intervention groups were invited to use the app as they wished, with the only suggestion of recording at least 3 sessions of a minimum of 10 minutes per week of physical education.

### Risk of Bias in Studies

The RoB assessment for the included RCT studies is graphically reported in Figure 2 using the Robvis tool [84]. The first study presents some concerns in the overall RoB, due to the randomization process since it is not clear how the experimenters performed it. Moreover, the study reports the registration of a protocol with a registration number, but in the mentioned database, it is not possible to find the protocol. In this case, it cannot be excluded that the results were not analyzed by a prespecified analysis plan that was finalized before unblinded outcome data were available for analysis. Indeed, the authors reported having several missing data but without any reasonable explanation.

The same 2 domains (D1 and D5) influence the overall RoB of the second study as well. The second study has the overall RoB indicated as “high risk.” Domains 2, 3, and 4 are at high RoB, and those are influencing the overall RoB. The corresponding authors of the studies were contacted, but no answer was ever received.

### GRADE Assessment

The assessment of the quality of evidence adopting the GRADE approach could not be performed due to the high heterogeneity of the outcomes considered in the studies included. First, the 2 studies did not consider the same outcome. Moreover, even involving the same sphere of interest (ie, physical activity) did not consider the same outcomes. Specifically, the first study [30] mainly considered physiological outcomes (ie, VO_2_ max, blood glucose), while the second study [31] investigated performance outcomes (eg, 20-m shuttle run test, handgrip). Therefore, it is not possible to assess the quality of this evidence.

## Discussion

### Principal Findings

The included studies were overall characterized by a high RoB due to many missing values, high dropout, small sample sizes, and poor data reporting. In particular, the randomization processes were evaluated with the RoB 2.0 tool with “some concerns” as this tool requires this scoring if the paper does not describe the randomization procedures adopted in detail but just mention their adoption. Additionally, for 1 study, a protocol was not available. Moreover, the RoB of the second study was influenced by the decision of not including in the data analysis the participants who had missing data in 1 of the evaluations or were outliers. Thus, it might not be excluded that the results were influenced.

Furthermore, their study designs were significantly different, and it was not possible to make a direct comparison between their results. Our findings highlight the urgent need for standardized outcome measures to enable the generation of stronger, comparable evidence in this field. For each health domain examined, a validated set of standardized outcomes should be developed, allowing for their consistent use across diverse study designs and settings, including RCTs. Such standardization would facilitate more accurate assessments of mobile app effectiveness on health outcomes in healthy adults. Due to the heterogeneity of outcome measures among the included studies, it remains difficult to draw definitive conclusions regarding the efficacy of health-related mobile apps in this population. Therefore, future studies should aim to include larger sample sizes to enhance statistical power and improve the reliability of findings. Additionally, greater participant numbers may also help mitigate issues related to dropout and incomplete data during interventions. Moreover, intervention times should also be standardized to be able to compare results from different studies. Another problem that emerges from our results is the lack of standardized apps or guidelines to develop them for different health domains, and this might be seen from the different outcomes considered in each study included in our review. A standardized version of the health app could allow for having a set of common health data across different apps, with the possibility of adding other health variables specific to each app based on its characteristics and aims. In this way, studies could compare the use of different health apps for the same domain and consider a minimum set of common health variables.

Other systematic reviews were carried out on mHealth in the last years [85-89] and were also characterized by a very limited number of selected studies with high heterogeneity, and therefore they could not assess mHealth effectiveness. Indeed, many of the studies they included presented mixed results of the delivered interventions and the way of delivering them [64,87,90,91]. Hence, those studies did not assess the effectiveness of the same intervention delivered with and without mHealth, as we conversely did in our work. Considering the available literature, it is fundamental to emphasize the need to evaluate the effectiveness of specific mHealth interventions. However, the intrinsic variability in the designs and the scarce quality of the currently available studies do not allow us to state if mobile apps can be effective to improve health variables.

To overcome this problem, we decided to include in our work the studies that compared the same intervention provided with and without the mobile app, and additionally also the studies considering the same treatment provided via mobile app against the treatment. By applying these severe criteria, many studies were excluded from this systematic review for improper control, leaving only 2 studies to analyze. Consistent with our findings, other works reported the need for more studies with clearer designs to test the effectiveness of mHealth technology in different settings [92-95].

### Studies Included

Digging into the included studies, Zongpa et al reported that physical activity–related variables improved over a 4-week period, specifically VO_2_ max, heart rate variability, and fasting blood glucose. It should be noted that dropouts were 11.32%, and VO_2_ max improvements may be questioned because the validity of a submaximal test in healthy people is questionable. Moreover, the VO_2_ max improvement reported by this study was only 1.33%, while the minimal clinically important difference for VO_2_ max should be higher than 6% [96]. Finally, the results should be taken carefully, as the results included in the analysis considered only an intervention adherence of at least 70%, thus imposing a possible bias in the selection of the results.

In the second study, Muntaner-Mas et al reported that many physical activity–related variables evaluated in the study improved, and the authors decided to split them into 3 categories: fatness indicators, physical fitness components, and self-reported fitness.

Starting from fatness indicators (ie, weight, waist, and hip circumference), no changes were obtained that could be attributable to the app. Physical fitness components (ie, 20-m shuttle run laps, handgrip strength, standing broad jump, 4×10 m shuttle run, and sit-and-reach) were improved, but even though few changes are indicated as statistically significant, the actual improvements are minimal and might not even be clinically relevant [97]. Second, improvements of a few units of centimeters or seconds obtained in 9 weeks and from a healthy and young population could be considered scarce. The category “self-reported physical fitness” explored 5 domains, and all the components were evaluated via Likert-type questions and reported the results obtained from the International Fitness Scale. The results improved for the intervention group and decreased for the control group, but considering the possible bias emerging from the self-evaluation, improvements should be carefully addressed since changes were minimal. Even in this category, the results on mobile app effectiveness could not be considered conclusive.

Despite the study considering the mobile app effective in improving physical activity–related variables, there were many missing data (ie, about 30%), and the sample size is limited. Summarizing, the effectiveness of the Vidahora app cannot be assessed. Although the selected studies highlighted that diet and physical activity levels can be improved by mHealth apps, their weak methodological design raises some concerns about their conclusions.

### Limitation

A limitation of this work must be acknowledged: this systematic review included only studies involving healthy participants. Therefore, it is not possible to report anything about people with chronic conditions or pathologies. Additionally, a librarian was not consulted to develop the research strings, as people with expertise in conducting systematic reviews, and in their methodology, were consulted.

### Conclusions

Despite the studies we included seeming to support the effectiveness of mobile apps to improve physical activity, diet-related variables, or their combination in healthy adults, their poor methodological quality as well as the high variability in literature does not allow any definitive conclusion on this topic. Besides, the long-term effects of mobile apps interventions on different outcomes are scarce [92,95], and further research is needed. In addition, the interventions’ (eg, activity, diet) effectiveness should be tested a priori and then provided via mHealth. Some urgent needs emerge from the literature analyzed and from this study. Specifically, for future studies, there is a need for high-quality RCT designs with large sample sizes to better assess the possible effects of health apps and the generalizability of results. Moreover, there is a need for clearer and consistent methodology that could provide stronger evidence of effectiveness, more transparent reporting of results that would prevent any bias and would additionally allow for acknowledging what is not working with apps for health and why, and addressing healthy people to test the mobile apps’ effectiveness in preventing diseases and improving health conditions. Furthermore, standardized outcomes for each health domain of interest (eg, physical activity, diet) should be adopted, allowing for comparing the results of different studies and populations. Additionally, different studies could include, in the same health domain of interest, the chosen standardized outcomes as well as new ones, to try to expand possible results. Moreover, mHealth should be tested and validated by both patients and users before using them to deliver an intervention. Further research should test mobile apps as a tool supporting preventive approaches for health and well-being in young people and healthy participants as well. Finally, clear guidelines should be created on how to build up different types of mHealth, specifically for mobile apps, to standardize this process among health apps and to further try to ensure better use of this technology in the active aging and well-being fields.

## Supplementary material

10.2196/66881 Multimedia Appendix 1 Reasons for exclusions (mobile apps for physical activity).

10.2196/66881 Multimedia Appendix 2 PRISMA (Preferred Reporting Items for Systematic Reviews and Meta-Analyses) flow diagrams.

10.2196/66881 Checklist 1 PRISMA checklist.

10.2196/66881 Checklist 2 PRISMA-S checklist.

## Abbreviations

GRADE: Grading of Recommendations, Assessment, Development and Evaluation
mHealth: mobile health
PRISMA: Preferred Reporting Items for Systematic reviews and Meta-Analyses
PRISMA-S: Preferred Reporting Items for Systematic Reviews and Meta-Analyses Literature Search Extension
RCT: randomized controlled trial
RoB: risk of bias
VO_2_ max: maximal oxygen consumption
