# Supplementary material for: Mobile Apps to Improve Health Parameters in Healthy Adults: Systematic Review
Source: JMIR Mhealth Uhealth. 2026 Jan 16;14:e66881. doi: 10.2196/66881 (PMC12810950; doi:10.2196/66881)
Supplement: Multimedia Appendix 1 [file mhealth-v14-e66881-s001.docx]

**Appendix B – Reasons for exclusions (Mobile apps for Physical Activity)**

| **Title** | **Authors** | **Reason for exclusion** |
| --- | --- | --- |
| A Contactless App-Based Intervention to Improve Health Behaviors in Airline Pilots: A Randomized Trial. | Wilson et al. | Wrong population |
| A mobile app-based intervention improves anthropometry, body composition and fitness, regardless of previous active-inactive status: a randomized controlled trial | Gómez-Cuesta et al. | Wrong population |
| A smartphone "app"-delivered randomized factorial trial targeting physical activity in adults. | Fanning et al. | Wrong population |
| A Social Networking and Gamified App to Increase Physical Activity: Cluster RCT. | Edney et al. | Wrong intervention |
| An-m-Health Intervention Using Smartphone App to Improve Physical Activity in College Students: A Randomized Controlled Trial. | Al-Nawaiseh | Wrong intervention |
| Are Smartphones Better in Guiding Physical Activity Among Sedentary Young Adults? A Randomised Controlled Trial | Tulasiram et al. | Wrong intervention |
| Combining Web-Based Gamification and Physical Nudges With an App (MoveMore) to Promote Walking Breaks and Reduce Sedentary Behavior of Office Workers: Field Study. | Mamede et al. | Wrong population |
| Effect of a Nine-Month Web- and App-Based Workplace Intervention to Promote Healthy Lifestyle and Weight Loss for Employees in the Social Welfare and Health Care Sector: A Randomized Controlled Trial | Balk-Møller et al. | Wrong population |
| Effect of Adding Telephone-Based Brief Coaching to an mHealth App (Stay Strong) for Promoting Physical Activity Among Veterans: Randomized Controlled Trial. | Damschroder et al. | Wrong population |
| Effectiveness of a 3-Month Mobile Phone-Based Behavior Change Program on Active Transportation and Physical Activity in Adults: Randomized Controlled Trial. | Ek et al. | Wrong intervention |
| Effectiveness of a smartphone application to promote physical activity in primary care: the SMART MOVE randomised controlled trial. | Glynn et al. | Wrong population |
| Effectiveness of an mHealth Application for Physical Activity Promotion Among Thai Older Adults: A Randomized Controlled Trial. | Pomkai et al. | Wrong population |
| Effectiveness of technology-assisted and self-directed interventions to sit less and move more among Indian desk-based office workers: A three-arm cluster randomised controlled trial (SMART-STEP trial). | Chandrasekaran et al. | Wrong population |
| Effectiveness of the Safe Step Digital Exercise Program to Prevent Falls in Older Community-Dwelling Adults: Randomized Controlled Trial. | Pettersson et al. | Wrong population |
| Effects of an mHealth physical activity intervention to prevent osteoporosis in premenopausal women. A randomized controlled trial. | Sanchez-Trigo et al. | Wrong population |
| Effects of Mobile Health Prompts on Self-Monitoring and Exercise Behaviors Following a Diabetes Prevention Program: Secondary Analysis From a Randomized Controlled Trial. | MacPherson et al. | Wrong population |
| Effects of peer support and mobile application-based walking programme on physical activity and physical function in rural older adults: a cluster randomized controlled trial | Cai et al. | Wrong population |
| Effects of Smartphone-Based Remote Interventions on Dietary Intake, Physical Activity, Weight Control, and Related Health Benefits Among the Older Population With Overweight and Obesity in China: Randomized Controlled Trial | Zhang et al. | Wrong population |
| Effects of Three Motivationally Targeted Mobile Device Applications on Initial Physical Activity and Sedentary Behavior Change in Midlife and Older Adults: A Randomized Trial. | King et al. | Wrong population |
| Efficacy of an m-Health Physical Activity and Sleep Health Intervention for Adults: A Randomized Waitlist-Controlled Trial. | Murawski et al. | Wrong population |
| Efficacy of Individualized Sensory-Based mHealth Interventions to Improve Distress Coping in Healthcare Professionals: A Multi-Arm Parallel-Group Randomized Controlled Trial. | Baumann et al. | Wrong intervention |
| Efficacy of mHealth-Based Workplace Health Promotion Strategy in Improving Cardiorespiratory Fitness in a Healthcare Setting: A Randomized Controlled Study. | Ghazala et al. | Wrong population |
| Evaluating Machine Learning-Based Automated Personalized Daily Step Goals Delivered Through a Mobile Phone App: Randomized Controlled Trial. | Zhou et al. | Wrong intervention |
| Evaluating Machine Learning-Based Automated Personalized Daily Step Goals Delivered Through a Mobile Phone App: randomized Controlled Trial | Zhou et al. | Wrong intervention |
| Evaluation of an mHealth intervention aiming to improve health-related behavior and sleep and reduce fatigue among airline pilots. | Van Drongelen et al. | Wrong population |
| Evaluation of stAPP: a smartphone-based intervention to reduce prolonged sitting among Belgian adults. | Arrogi et al. | Wrong intervention |
| Examining mediators of intervention efficacy in a randomised controlled m-health trial to improve physical activity and sleep health in adults. | Murawski et al. | Wrong population |
| Fitness mobile apps positively affect attitudes, perceived behavioral control and physical activities | Gabbiadini et al. | Wrong population |
| Follow-up monitoring of physical activity after rehabilitation by means of a mobile application: Effectiveness of measurements in different age groups. | Saran et al. | Wrong intervention |
| Gamifying Accelerometer Use Increases Physical Activity Levels of Sedentary Office Workers. | Gremaud et al. | Wrong intervention |
| Increasing the Effectiveness of a Physical Activity Smartphone Intervention With Positive Suggestions: Randomized Controlled Trial. | Skvortsova et al. | Wrong intervention |
| Living labs for a mobile app-based health program: effectiveness of a 24-week walking intervention for cardiovascular disease risk reduction among female Korean-Chinese migrant workers: a randomized controlled trial. | Kim et al. | Wrong intervention |
| Long-term Effects of the Use of a Step Count-Specific Smartphone App on Physical Activity and Weight Loss: Randomized Controlled Clinical Trial. | Yoshimura et al. | Wrong population |
| mActive: A Randomized Clinical Trial of an Automated mHealth Intervention for Physical Activity Promotion. | Martin et al. | Wrong population |
| mHealth to Support Outdoor Gym Resistance Training: The ecofit Effectiveness RCT | Plotnikoff et al. | Wrong intervention |
| Multiple behavior changes in diet and activity: a randomized controlled trial using mobile technology | Spring et al. | Wrong population |
| Personalized digital behaviour interventions increase short-term physical activity: a randomized control crossover trial substudy of the MyHeart Counts Cardiovascular Health Study. | Javed et al. | Wrong population |
| Short- and Long-term Effects of a Mobile Phone App in Conjunction With Brief In-Person Counseling on Physical Activity Among Physically Inactive Women: The mPED Randomized Clinical Trial | Fukuoka et al. | Wrong population |
| Smartphone- and Paper-Based Delivery of Balance Intervention for Older Adults Are Equally Effective, Enjoyable, and of High Fidelity: A Randomized Controlled Trial. | Lugade et al. | Wrong intervention |
| The Effect of a Future-Self Avatar Mobile Health Intervention (FutureMe) on Physical Activity and Food Purchases: Randomized Controlled Trial. | Mönninghoff et al. | Wrong population |
| The effect of digital physical activity interventions on daily step count: a randomised controlled crossover substudy of the MyHeart Counts Cardiovascular Health Study. | Shcherbina et al. | Wrong population |
| The Effect of the ERVE Smartphone App on Physical Activity, Quality of Life, Self-Efficacy, and Exercise Motivation for Inactive People: a Randomized Controlled Trial | Gür et al. | Wrong intervention |
| The efficacy of online physical activity interventions with added mobile elements within adults aged 50 years and over: Randomized controlled trial. | Collombon et al. | Wrong population |
| The impact of a Web-based app (eBalance) in promoting healthy lifestyles: randomized controlled trial. | Safran Naimark et al. | Wrong population |
| TRACK & ACT: a pragmatic randomised controlled trial exploring the comparative effectiveness of pedometers and activity trackers for changing physical activity and sedentary behaviour in inactive individuals | Ryan et al. | Wrong intervention |
| Training and Detraining Effects of a Physical Activity Program Implemented Through Mobile Applications in Adolescents | Mateo-Orcajada et al. | Wrong study design |
| Using a Fitbit-based Walking Game to Improve Physical Activity Among U.S. Veterans. | Simmering et al. | Wrong population |
| Weight loss maintenance among truck drivers in the SHIFT randomised controlled trial, USA. | Olson et al. | Wrong population |
| Which behaviour change techniques are effective to promote physical activity and reduce sedentary behaviour in adults: a factorial randomized trial of an e- and m-health intervention. | Schroé et al. | Wrong intervention |
