# Supplementary material for: Mobile Apps to Improve Health Parameters in Healthy Adults: Systematic Review
Source: JMIR Mhealth Uhealth. 2026 Jan 16;14:e66881. doi: 10.2196/66881 (PMC12810950; doi:10.2196/66881)

**Appendix A – PRISMA flow diagrams**

***Figure 2 - PRISMA for Mobile Apps for Diet***


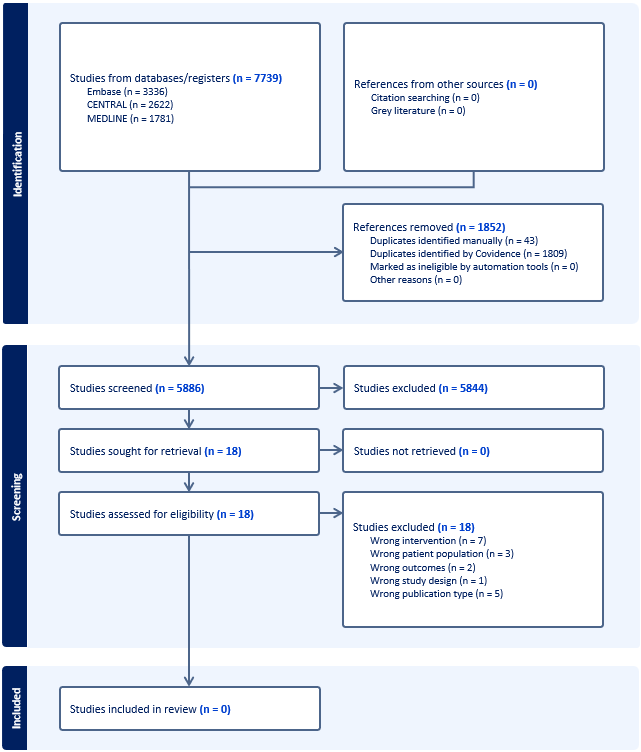


***Figure 3 – PRISMA flow diagram for Mobile Apps for Physical Activity and Diet***


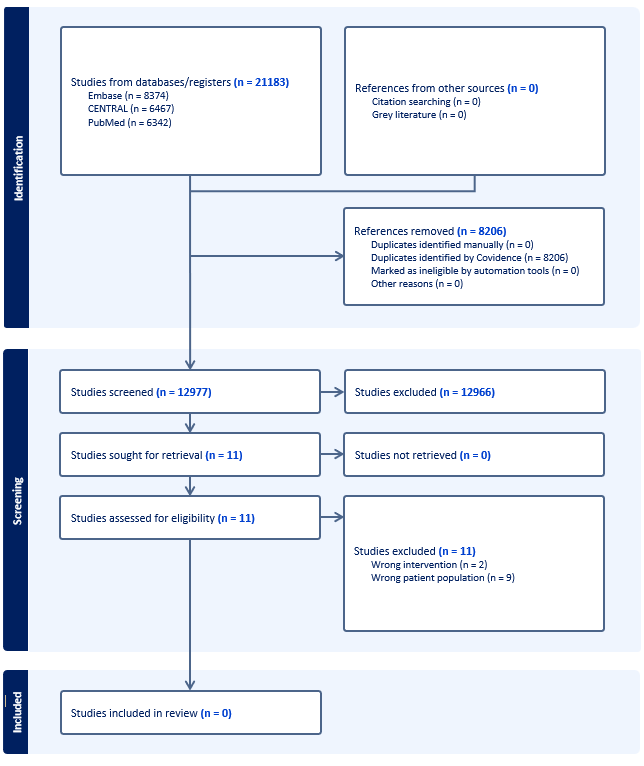

Supplement: Multimedia Appendix 2 [file mhealth-v14-e66881-s002.docx]
